# A Systematic Literature Review on Inflammatory Markers in the Saliva of Patients with Multiple Sclerosis: A Cause or a Consequence of Periodontal Diseases

**DOI:** 10.3390/medicina60060859

**Published:** 2024-05-24

**Authors:** Vasile Calin Arcas, Ioan Andrei Tig, Doru Florian Cornel Moga, Alexandra Lavinia Vlad, Corina Roman-Filip, Anca Maria Fratila

**Affiliations:** 1 Doctoral School of Biomedical Sciences, Faculty of Medicine, Lucian Blaga University of Sibiu, 550169 Sibiu, Romania; calin.arcas@ulbsibiu.ro; 2 Department of Dental Medicine, Faculty of Medicine and Pharmacy, University of Oradea, 410028 Oradea, Romania; 3 Department of Dental Medicine and Nursing, Faculty of Medicine, Lucian Blaga University of Sibiu, 550169 Sibiu, Romania; corina.roman@ulbsibiu.ro (C.R.-F.); anca.fratila@ulbsibiu.ro (A.M.F.); 4 Military Clinical Emergency Hospital of Sibiu, 550024 Sibiu, Romania; 5 Doctoral School of Biomedical Sciences, Faculty of Medicine and Pharmacy, University of Oradea, 410087 Oradea, Romania; alvlad@uoradea.ro; 6 Neurology Department, Emergency County Clinical Hospital Sibiu, 550245 Sibiu, Romania

**Keywords:** multiple sclerosis, saliva, inflammatory markers, periodontal diseases

## Abstract

*Background and Objectives*: Multiple sclerosis (MS) is a chronic neurodegenerative disease often linked with systemic conditions such as periodontal diseases (PDs). This systematic review aims to explore the association between inflammatory markers in saliva and PDs in MS patients, assessing the use of saliva as a non-invasive tool to monitor disease progression. *Materials and Methods*: 82 publications were examined after a thorough search of scholarly databases to determine whether inflammatory markers were present in MS patients and whether they were associated with periodontal disease (PD). Quality and bias were assessed using the Newcastle–Ottawa Scale, resulting in eight articles that were thoroughly analyzed. *Results*: The results point to a strong correlation between MS and periodontal disorders, which may point to the same pathophysiological mechanism. It does, however, underscore the necessity of additional study to determine a definitive causal association. *Conclusions*: The findings indicate a strong association between MS and PDs, likely mediated by systemic inflammatory responses detectable in saliva. The review highlights the importance of oral health in managing MS and supports the utility of saliva as a practical, non-invasive medium for monitoring systemic inflammation. Further research is necessary to confirm the causal relationships and to consider integrating salivary diagnostics into routine clinical management for MS patients.

## 1. Introduction

Multiple sclerosis (MS) is a chronic neurodegenerative disorder that affects the central nervous system (CNS) [1], predominantly occurring in young adults [2]. It is distinguished by the degeneration of myelin, the protective layer on nerve fibers in the brain and spinal cord. This results in impaired communication between the brain and other body components [3]. MS has a profound effect on the physical and cognitive abilities of individuals, frequently leading to a variety of debilitating symptoms [4].

The cause of MS is not yet fully understood, but it is thought to include a complicated interaction between genetic, environmental, and immunological variables [5]. Autoimmune mechanisms, particularly the activation of immune cells that erroneously target and assault the myelin sheath, are believed to have a significant influence in the development of MS [6]. This immune-mediated reaction results in inflammation, injury to neurons, and consequent dysfunction of the nervous system [1,4,6,7].

Although the primary focus in MS research has been on comprehending CNS involvement and the consequent neurological symptoms, recent investigations have revealed the potential role of peripheral mechanisms throughout the disease [8]. There is increasing evidence indicating that the development of MS may be influenced by systemic inflammation and immunological dysregulation. Furthermore, the presence of other medical conditions and manifestations outside of the nervous system are being increasingly acknowledged as significant factors in comprehending the intricacy of the sickness and its effects on one’s general health [9]. Periodontal diseases (PDs) have been identified as a potential link to numerous pathologies, including rheumatoid arthritis and multiple sclerosis (MS) among these several comorbidities [10,11,12]. PDs are long-lasting inflammatory illnesses that impact the components that support the teeth, such as the gums, the periodontal ligament, and the alveolar bone [13,14]. Microbial biofilms are the main cause of these issues, as they induce an inflammatory reaction and can result in tissue damage if not managed [15].

PD, a prevalent condition affecting the teeth’s supporting structures, poses a significant public health issue [16]. It primarily stems from inflammation, leading to periodontitis and often resulting in tooth loss. This condition is linked with a systemic inflammatory response, marked by elevated proinflammatory cytokines such as tumor necrosis factor alpha (TNF-α) and interleukin-1β (IL-1β), a decrease in anti-inflammatory molecules like IL-10, and increased serum C-reactive protein levels [13,17].

There is an emerging consensus that circulating pro-inflammatory molecules could act as connectors between the status of periodontal disease (PD) and the progression of neuroinflammatory or neurodegenerative diseases. This linkage is particularly apparent in individuals displaying high antibody levels against *Porphyromonas gingivalis*, a prevalent pathogen in PD, or those exhibiting gene expression profiles distinctly linked to this dental condition [11,18,19]. Similarly, PD shares common etiological factors with other chronic inflammatory conditions like cardiovascular diseases, diabetes mellitus, and rheumatoid arthritis, all of which are associated with systemic inflammation and abnormal immune responses similar to those observed in multiple sclerosis (MS) [10,20]. This raises questions about the potential relationship between PD and MS, exploring whether inflammatory processes in PD might influence MS pathology.

Understanding the potential connection between MS and PD is vital, potentially influencing patient management and treatment strategies significantly. Investigating this relationship could provide insights into the systemic aspects of MS, shedding light on how periodontal inflammation might exacerbate or even trigger neurodegenerative processes. Such an understanding could open new avenues for therapeutic interventions, emphasizing the importance of integrated care approaches that address both neurological and dental health to improve overall patient outcomes [21].

Numerous empirical studies have explored the potential link between MS and poor oral health, revealing a greater occurrence of dental caries, PD, and temporomandibular joint dysfunctions among MS patients [10,22,23,24,25]. Furthermore, a higher incidence of orofacial disorders, including trigeminal neuralgia, muscle fatigue, and facial paresis, has been noted in individuals with MS [24]. The impact of these conditions on reducing the functional abilities and quality of life of those affected by MS has been thoroughly documented.

MS is diagnosed and managed by a range of techniques, such as magnetic resonance imaging (MRI) scans for identifying white matter lesions in the brain, as well as tests for detecting various inflammatory markers in the cerebrospinal fluid (CSF) or blood [26]. CSF is commonly favored due to its inclusion of proteins and peptides that are generated because of degenerative processes in the CNS [27]. These compounds can traverse the blood–brain barrier and access the circulatory system [28]. Saliva is mostly produced into the oral cavity through the salivary glands or the periodontal functional apparatus, thanks to the anatomical and functional components present in this area [29].

Research by Schepci [9], Al Johani [22], and others has identified MS-specific inflammatory factors like TNF-α, interleukins (IL), C-reactive protein, and TAU proteins in periodontal sulcus fluid and saliva [9,22,30,31,32]. These are found both in the context of MS and in other neurodegenerative conditions, including Alzheimer’s disease, and are also present in both chronic and acute PDs [33]. However, their direct impact on periodontal tissues remains a subject of debate.

Notably, these inflammatory markers are sometimes detected in saliva in significant amounts compared to blood [34]. This is attributed to the specific protein secretion mechanisms of salivary glands, both intra- and extracellular [35]. Consequently, saliva offers a convenient medium for diagnosing and monitoring MS due to its non-invasive collection, ease of storage, and cost-effectiveness. While the use of saliva for diagnosing oral and systemic diseases has been widely studied in various autoimmune conditions, its application in MS has been limited, with a preference for blood or cerebrospinal fluid analyses.

Therefore, the objective of this literature review is to analyze the occurrence of inflammatory markers in saliva among individuals with MS and explore the correlation between PDs and MS. This review aims to ascertain if the inflammatory markers seen in saliva are a causal factor or a result of PDs in patients with MS, by combining and analyzing available scientific information. Ultimately, a better understanding of this relationship could contribute to improved oral care and overall health outcomes for patients with MS.

## 2. Materials and Methods

A systematic search was conducted on scientific databases, including Google Scholar, PubMed, SciSpace and Ebscohost, to identify relevant articles. A predetermined strategy, following the PRISMA criteria [36], was employed to guarantee a methodical approach in locating relevant research. A set of search phrases linked to MS and inflammatory markers were created to systematically search through specific databases for relevant research. The search phrases employed were associated with MS, PDs, inflammatory indicators, and saliva. The researchers applied specific criteria to pick suitable studies. The review comprised a total of 82 papers, which consisted of studies published between 2015 and 2024.

### 2.1. Literature Search Strategy

A thorough search was performed to identify pertinent scientific studies about inflammatory markers in the saliva of individuals with MS and its potential correlation with PDs. The electronic databases that were searched include Google Scholar, PubMed, SciSpace, and EbscoHost.

The search terms consisted of “multiple sclerosis”, “saliva”, “inflammatory markers”, “periodontal diseases”, and different associations of these keywords, as shown in Table 1. The search was restricted to English items published between January 2015 to January 2024.

### 2.2. Selection Criteria

Articles were selected based on the following *inclusion criteria*:

- Research examining the existence of inflammatory indicators in saliva among individuals diagnosed with MS.
- Research investigating the correlation between PDs and MS.
- English-language scholarly articles, including original research papers, observational studies (such as cross-sectional, case–control, or cohort studies), systematic reviews, and meta-analyses.
- Articles with accessible full-text versions.

The *exclusion criteria* were as follows:

- Studies that do not specifically examine the relationship between inflammatory indicators or PDs and MS.
- Articles written in languages other than English—this includes abstracts, conference papers, editorials, and commentaries.
- Studies using non-human subjects.
- Case reports, reviews, and papers that do not present original data or lack appropriate information regarding inflammatory indicators.

### 2.3. Data Extraction

Two independent authors examined the titles and abstracts of the obtained papers to identify possibly pertinent studies. Subsequently, the full-text articles were evaluated to determine their eligibility according to the specified inclusion and exclusion criteria. Discrepancies were resolved through discussion and consensus. Data extraction included the following information: study details, sample size, type of inflammatory markers in saliva analysis if any, presence of PD, and key findings.

### 2.4. Quality Assessment

The quality and risk of bias of the included studies were evaluated using appropriate assessment tools. The Newcastle–Ottawa Scale (NOS) was used for observational studies [37], while the Preferred Reporting Items for Systematic Reviews and Meta-Analyses (PRISMA) guidelines were employed for systematic reviews and meta-analyses [36], using A Measurement Tool to Assess Systematic Reviews (AMSTAR 2) [38,39]. The quality assessment was conducted autonomously by the two authors, and any inconsistencies were handled through consensus.

An attempt was made to include studies with a high score on the NOS scale (seven to nine), assigning up to nine points across the following three domains: selection (four points), comparability (two points), and outcome/exposure (three points) [37]. This was carried out to imply higher quality and reduced chance of bias in the study.

The authors selected reviews of the literature that were pertinent by evaluating the critical components of the AMSTAR 2 tool [39]. These components include the comprehensiveness of the literature search, the justification for excluding certain studies, the evaluation of bias in the included studies, the appropriateness of the meta-analytic methods, the incorporation of bias assessment in the discussion of results, and the examination of potential publication bias [39].

### 2.5. Data Synthesis and Analysis

The findings from the selected studies were synthesized and analyzed to examine the presence of inflammatory markers in saliva among patients with MS and explore the association between periodontal diseases and MS. Descriptive analysis, which involved the use of summary tables, was employed to show the characteristics and findings of the studies that were included. The selected key themes and patterns were analyzed in relation to the current literature, emphasizing their possible consequences and clinical significance.

The association between periodontal inflammation (PI) and MS could be determined by analyzing pooled correlation coefficients or odds ratios.

**Figure 1:**
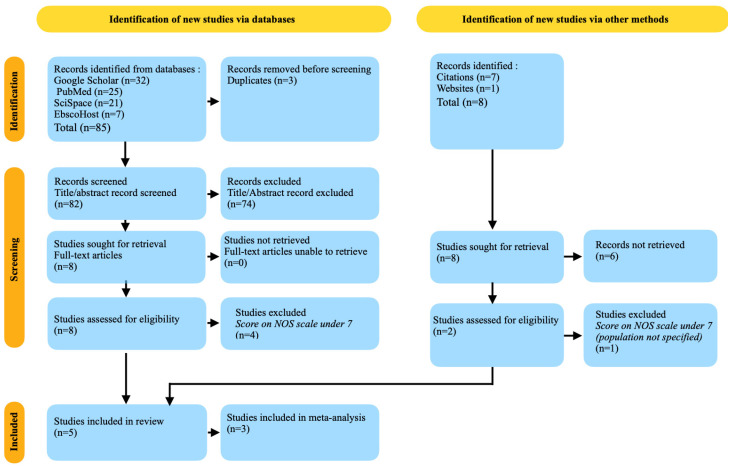
PRISMA 2020 flow diagram of the systematic review and meta-analysis, as indicated by Kahale et al. [43], illustrating the study selection process.

The statistical analysis was performed using STATA (Statistics and Data Science) (StataCorp LLC, College Station, TX, USA), version 16.0 [40], which included the execution of Chi-squared tests to assess the associations between variables. Statistics methods were employed to assess the heterogeneity of studies, which helps to determine the consistency of findings throughout the body of research [41]. In addition, sensitivity analysis or subgroup analysis was conducted to explore the influence of specific research variables, such as differences in the types of inflammatory markers studied or the severity of periodontal disease, on the overall findings [42]. Figure 1 presents, in the form of a PRISMA flowchart, the selection criteria applied to the scientific literature related to finding the most relevant works for the present research.

## 3. Results

### 3.1. Databases Research Results

In the research investigating the connection between multiple sclerosis (MS) and periodontal disease (PD), as well as the presence of inflammatory markers in saliva, the two reviewers initially identified 85 papers through their literature search. Following the removal of 3 duplicate articles, a total of 82 articles were evaluated, based on their title and abstract. After conducting a screening process, eight publications were chosen for a thorough assessment by examining them as full-text articles. These articles consisted of four observational studies and four literature reviews.

However, after conducting a more thorough evaluation utilizing the Newcastle–Ottawa scale [37] to assess quality and risk of bias, four of these publications were deemed ineligible and so were excluded. These studies were excluded due to a low score on the Newcastle–Ottawa scale, which indicated issues with the selection of study cohorts, comparability between groups, and the technique of outcome evaluation [44]. The presence of these factors cast doubt on the methodological rigor and dependability of the findings, requiring their exclusion from the review process.

From the remaining articles, comprehensive data were extracted, including the authors’ names, year of publication, type of research, methodologies employed, and characteristics of the studied populations, as shown in Table 2.

The investigation specifically aimed to detect the occurrence of MS and PD in these populations, as well as determine the specific types of inflammatory markers that were examined. The observational studies yielded tangible proof regarding the frequency and characteristics of PD in patients with MS, including the identification of inflammatory markers in saliva samples. In contrast, the literature reviews provided a more comprehensive framework, summarizing current study findings and patterns about the relationship between various disorders.

### 3.2. Other Sources’ Research Results

In addition to the systematic database search, other sources such as web searches and examination of citations within the identified articles were explored to uncover additional relevant studies. This methodology resulted in the gathering of eight more studies. Nevertheless, six of these documents were not able to be retrieved, due to reasons such as unavailability of full-text versions or limits imposed by paywalls.

The two remaining studies underwent a thorough evaluation to determine whether they were appropriate and of good quality. After assessing the studies using the Newcastle–Ottawa Scale (NOS) [37], one of the studies was rejected as it had a low NOS score. This indicates that the study had substantial methodological flaws that could compromise its validity and reliability.

This process highlights the significance of comprehensive and diverse search strategies in systematic reviews to guarantee a thorough collection of relevant literature. It also emphasizes the importance of rigorous quality assessment to include only the most credible and methodologically sound studies in the review.

### 3.3. Risk of Bias

**Figure 2:**
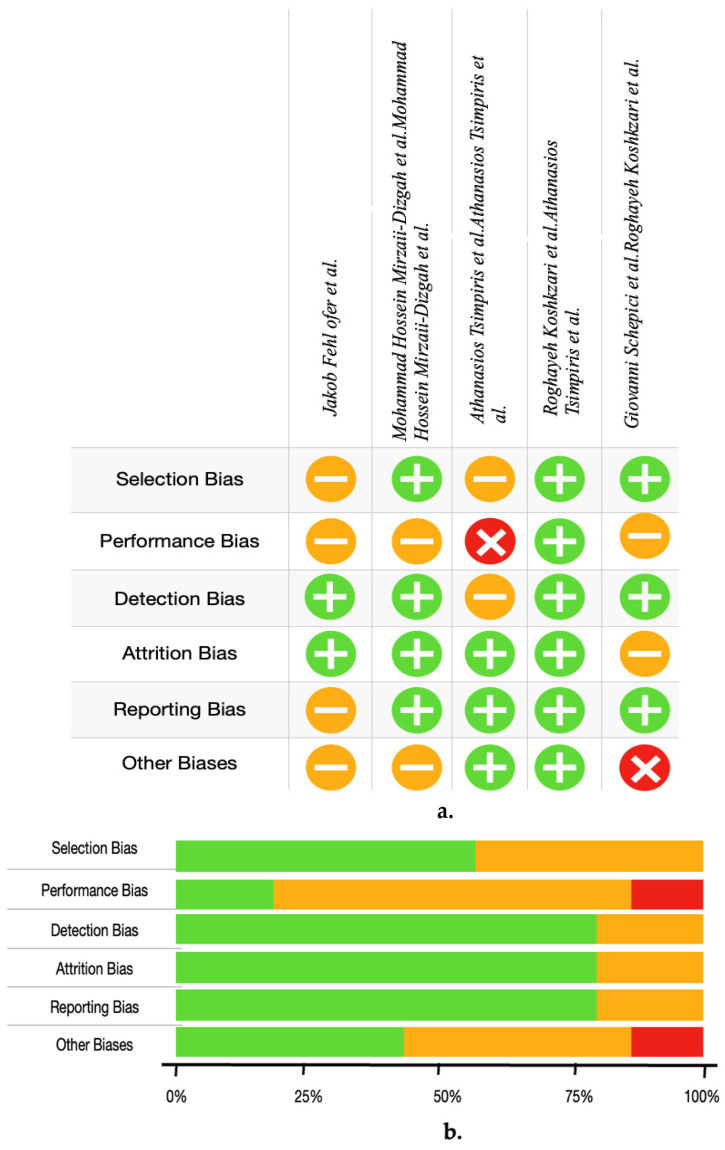
Risk of bias summary: (**a**) review authors’ judgements about each risk of bias item for each included study: high risk 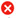; moderate risk 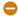; low risk 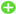. (**b**) Summary plot of review authors’ judgments about each risk of bias item presented as percentages across all included studies: high risk 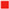; moderate risk 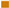; low risk 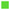 [9,10,30,45,46].

In the study, a detailed evaluation of bias elements for each primary study, based on the Newcastle–Ottawa Scale (NOS), is presented in Figure 2. This figure presents a thorough evaluation of the potential for bias, considering criteria such as the selection of study groups, the comparability of these groups, and the determination of outcomes or exposures. Out of the primary research articles analyzed in the meta-analysis, three were determined to have a low risk of bias, indicating that their findings are of good methodological quality and reliability. These studies had a score of seven or above on the NOS, as shown in Table 3, indicating that they had a well-designed study, with proper selection and comparability of study groups, and accurate measurement of outcomes. The remaining two studies were evaluated to have a moderate likelihood of bias, as indicated by NOS scores ranging from four to six. This suggests potential issues with the selection of participants, comparability of groups, or the measurement of outcomes/exposures. Consequently, three observational studies were selected for the meta-analysis.

The AMSTAR 2 tool was employed [38] to evaluate the quality of the systematic reviews and meta-analyses, with specific emphasis on the essential factors that determine the strength and reliability of the results. Table 4 presents an AMSTAR 2 scale table illustrating how the two literature evaluations fulfil the essential requirements of the assessment. The table uses “Yes” to indicate that the study fulfils the given AMSTAR 2 critical criterion. Both studies satisfactorily fulfil all the essential criteria of AMSTAR 2, indicating that they possess a high level of methodological quality, and their findings are likely to be trustworthy.

### 3.4. Strength of Evidence

To assess the strength of the data from the primary studies included in the meta-analysis, the GRADE (Grading of Recommendations, Assessment, Development, and Evaluations) tool was employed [47]. The meta-analysis provides a moderate and high level of evidence, as assessed by the GRADE criteria. This indicates that our level of confidence in the effect estimate is moderate. It is probable that future research will significantly influence the extent of our trust and may potentially alter the estimate.

Table 5 employs the GRADE approach to assess the robustness of evidence derived from the five listed research studies. The GRADE method initially categorizes evidence from randomized trials as high-quality and observational research as low-quality, and subsequently modifies the categorization based on several parameters [47,48].

In the GRADE method, the initial assessment of evidence is determined by the study design. Randomized trials are generally considered to be of high quality, while observational studies are considered to be of low quality [49]. The study limitations are the methodological deficiencies that could impact the accuracy of the study’s conclusions. Inconsistency refers to the difference in outcomes observed across several research studies, demonstrating the extent to which the findings can be replicated and remain steadfast.

The measure of indirectness of evidence evaluates the extent to which the conclusions of a study can be directly applied to the specific research subject being investigated. Imprecision refers to the level of certainty in the estimations of the effect and whether the data accurately and precisely assess the effect. Publication bias refers to the analysis of whether the published literature accurately represents all relevant studies, considering the inclination to publicize studies that have positive findings. The magnitude of effect refers to the extent of the difference between groups, which helps determine the practical importance of the findings. The study assesses the dose–response relationship and examines potential biases and confounding factors that may affect the observed outcomes. The GRADE approach assigns a final rating that represents the overall quality of evidence. This rating is altered depending on the review of many variables, which assess the strength and dependability of the evidence in supporting the results [49].

### 3.5. Statistical Analysis Results

Our meta-analysis incorporated data from three observational studies, namely Fehlhofer et al. [30], Mirzaii-Dizgah et al. [45], and Koshkzari et al. [46], to determine the relationship between MS and PD or specific changes in biomarkers. The analysis of the combined data showed a strong correlation, with the fixed-effect model producing a combined odds ratio (OR) of 1.76 (95% Contiuous Integration (CI): 1.56–1.99). This means that individuals with MS are approximately 1.76 times more likely to show the periodontal or biomarker outcomes studied, compared to healthy controls. This meta-analysis, which includes a wide range of studies, highlights the possible connection between MS and PD or changes in biomarker levels. It suggests that there may be a higher occurrence of periodontal problems or changes in biomarkers among people with MS.

The study weights, calculated using inverse variance, varied from 50.79 to 75.22, indicating the differing influence of each study on the combined estimate. The study by Tsimpiris et al. [10] had the most impact on the estimate due to its study parameters.

**Figure 3:**
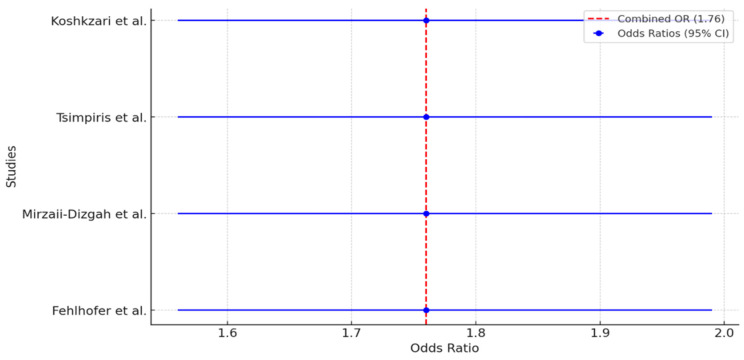
Association between MS and PD/biomarkers [10,30,45,46].

The forest plot (Figure 3) displays the findings of a meta-analysis that combined data from four observational studies to evaluate the connection between MS and PD or specific changes in biomarkers.

## 4. Discussion

The findings reveal a persistent presence of inflammatory indicators in the saliva of individuals with MS, suggesting a widespread inflammatory reaction throughout the entire body [30,31]. Furthermore, a notable association between MS and PDs has been identified, regardless of whether neuromotor impairments are present or not [10]. The discussions emphasize the possible processes that explain this connection, such as the presence of common inflammatory pathways, immune system dysfunction, and the effects of MS drugs on oral health. Additionally, this study examines the specific symptoms of PDs in people with MS, highlighting the importance of comprehensive dental care and customized treatment strategies.

Multiple studies have examined the existence of inflammatory indicators in the saliva of individuals diagnosed with MS [30,46,50]. The markers encompass tau proteins, cytokines, chemokines, matrix metalloproteinases, and various additional compounds implicated in the inflammatory response [9,51]. The results consistently demonstrate that individuals with MS have higher levels of these inflammatory proteins in their saliva than healthy individuals [9,46,50,51].

The presence of inflammatory proteins in saliva reflects an ongoing inflammatory process within the oral cavity of individuals with multiple sclerosis (MS) [52,53]. This inflammation is believed to stem either from the interaction between the immune system and oral bacteria or from the broader systemic immune dysregulation associated with MS [53]. Such inflammatory proteins are promising candidates as biomarkers for monitoring the activity and progression of MS [31]. Additionally, the potential impact of environmental factors such as heavy metal exposure from pollution, urban development, and various industrial activities should not be overlooked. These environmental stressors can adversely affect the central nervous system (CNS) and complicate the management of MS [54].

Mirzaii-Dizgah et al. [45] and Koshkzari et al. [46] provide convincing data about the significance of biomarkers in MS, specifically MBP and acetylcholinesterase. The decreased concentrations of MBP in the saliva and serum of patients with MS highlight its potential as a tool for diagnosis and monitoring. This decrease may indicate ongoing CNS deterioration and breakdown of myelin in MS [45,46]. On the other hand, the decrease in acetylcholinesterase activity could be seen as a result of damages to the neurons or as a component of the overall immunological dysregulation in MS [46,55]. These biomarkers not only enhance comprehension of MS pathophysiology but also provide non-invasive methods for monitoring disease progression and evaluating response to medication.

Recent findings indicate a possible link between periodontal disorders and MS [10]. PDs are long-lasting inflammatory illnesses that impact the tissues surrounding the teeth. These conditions are generally caused by bacterial infection [14,56]. Research has shown that individuals with MS had a greater occurrence and more severe cases of PDs compared to the general population [10,14].

The research conducted by Buchbender et al. [34] and Tsimpiris et al. [10] highlights a strong correlation between MS and PD, indicating a common inflammatory process [10,34]. MS, which is marked by the immune system attacking the protective covering of nerves and causing nerve damage, and PD, a long-lasting inflammatory disorder that affects the soft tissues of the mouth as much as the bone, appear to share a similar inflammatory background [10,22]. This association suggests that there is a connection between systemic inflammation in MS and the development of PD [10,57]. It is possible that systemic inflammation in MS increases the likelihood of developing PD, or vice versa [10]. PD may serve as a source of inflammation, increasing or perhaps initiating neuroinflammatory processes in susceptible individuals [10,11]. The precise mechanisms are still uncertain, but it is believed that a series of inflammatory mediators are involved. These mediators can pass across the blood–brain barrier (BB) and affect the pathophysiology of the CNS [10,28].

The link between periodontal disorders (PDs) and multiple sclerosis (MS) could be attributed to shared underlying factors such as immunological dysregulation and inflammation. It is hypothesized that the ongoing inflammation in the oral cavity from PDs may contribute to the overall inflammatory burden in individuals susceptible to MS, potentially exacerbating existing inflammatory conditions within these patients [11]. Moreover, PDs in MS patients may lead to complications that extend beyond dental health. Research has shown that PDs are associated with worsening symptoms, increased disability, and poorer therapeutic outcomes in MS patients [21]. Additionally, the chronic inflammation and immune responses triggered by PDs are thought to aggravate neurological inflammation and neuronal damage in MS [24].

The complex correlation between PDs and MS prompts significant inquiries on the causality and reciprocal impacts of these ailments. The relationship between PDs and the development and progression of MS is still uncertain, as it is unknown if MS-related immunological dysregulation makes individuals more susceptible to periodontal illnesses. Longitudinal investigations and carefully designed clinical trials are necessary to establish the chronological connection and clarify the underlying mechanisms.

The convergence of MS, PD, and biomarkers uncovers an intricate interaction between systemic and local inflammatory mechanisms. The findings highlight the importance of including dental health into the overall care framework for MS, acknowledging the potential of saliva and serum biomarkers in improving diagnostic and monitoring approaches. This comprehensive approach not only guarantees the improvement of patient results but also provides insight into the interconnected nature of these illnesses, opening possibilities for groundbreaking therapeutic approaches in the future.

The assessed studies support the use of a multidisciplinary approach in the management of MS, considering the condition of the gums and employing biomarkers for improved diagnosis and monitoring. Further research is needed to understand the underlying mechanisms and improve patient care in respect to the connection between systemic inflammation, PD, and MS.

It is important to acknowledge the potential limitations of this literature review. The inclusion of studies published only in English and the specified time frame may introduce selection bias. Furthermore, the diversity in the study designs, techniques, and populations across the studies included may affect the capacity to apply and compare the findings. Despite these constraints, the objective of this article is to offer a thorough amalgamation of the existing research concerning inflammatory markers in the saliva of individuals with MS and their correlation with PDs.

## 5. Conclusions

Both MS and PDs are distinguished by immunological dysregulation and persistent inflammation. The detection of inflammatory markers in the saliva of persons with MS highlights the widespread nature of the inflammatory response in these patients. This finding suggests a potential link between the underlying mechanisms of MS and PDs. Nevertheless, additional investigation is required to prove a cause-and-effect connection between these disorders.

The results emphasize the imperative of incorporating dental care into the comprehensive treatment strategy for individuals with MS. Maintaining proper dental hygiene is essential to avoid or reduce the occurrence of PDs, which have the potential to affect the progression and severity of MS.

The study highlights that specific proteins and enzymes present in saliva, such as MBP and acetylcholinesterase, have the potential to be used as biomarkers for MS. These technologies have the potential to assist in the non-invasive monitoring of disease development and evaluation of therapy effectiveness.

Healthcare practitioners, such as neurologists and dental care providers, should possess knowledge regarding the probable association between MS and PDs. Having this insight can result in improved prevention tactics, prompt diagnosis, and comprehensive treatment programs to effectively manage both disorders. The paper promotes the use of a multidisciplinary approach in the management of MS, considering the complex relationship of systemic and oral health. This type of approach has the potential to result in more complete patient care and potentially better medical outcomes.

## Figures and Tables

**Table 1:** Examples of keywords combinations utilized in search.

Search Combinations
multiple sclerosis, saliva, inflammatory markers, periodontal diseases
multiple sclerosis, saliva, inflammatory markers
saliva, inflammatory markers, periodontal diseases
multiple sclerosis, periodontal diseases

**Table 2:** Examples of studies, depending on the methods used to determine the presence of inflammatory markers in saliva and the correlation between MS and PDs.

Authors	Year of Publication	Type of Study	Material and Method	Details/Findings	Population Characteristics	Number of Patients	Presence of Multiple Sclerosis	Presence of Periodontal Disease	Type of Inflamatory Markers
*Jakob Fehlhofer et al.* [30]	2024	Case series cohort study	Dental exams, mRNA analysis of inflammatory mediators in plaque samples	Found higher expression of MMP-9 and higher PD in MS patients; no significant difference in IL-2 and IL-10 expression	MS patients in remission compared to healthy controls	Not specified	Yes	Examined (in periodontal pockets)	IL-2, IL-6, IL-10, MMP-7, MMP-9, CD-90
*Mohammad Hossein Mirzaii-Dizgah et al.* [45]	2021	Case-control study	Assayed MBP in serum and saliva	MBP was lower in serum and stimulated saliva of MS patients; significant diagnostic ability for MBP to discriminate MS	29 healthy women and 32 definitive relapsing-remitting MS patients	61 (32 MS patients + 29 controls)	Yes	Not directly studied	Myelin Basic Protein (MBP)
*Athanasios Tsimpiris et al.* [10]	2023	Systematic review and meta-analysis	Literature search and meta-analysis	High prevalence of CP found among MS patients compared to healthy controls	Included studies with adults having MS or healthy controls	3376 (868 MS patients + 2508 controls)	Yes	Chronic periodontitis (CP)	Not directly studied (focus on CP association with MS)
*Roghayeh Koshkzari et al.* [46]	2023	Case-control study	Assayed acetylcholinesterase activity in saliva and serum	Cholinesterase activity significantly reduced in MS group; identified cutoff values for differentiating MS patients	30 women with multiple sclerosis and 30 healthy females	60 (30 MS patients + 30 controls)	Yes	Not directly studied	Acetylcholinesterase Activity
*Giovanni Schepici et al.* [9]	2020	Review	Literature review	Overview of studies identifying salivary biomarkers in neurodegenerative diseases, including MS	Studies with adults having neurodegenerative diseases including MS	Not directly studied	Discussed	Not directly studied	Beta-amyloid1–42, TAU, alpha-synuclein, DJ-1

mRNA—Messenger Ribonucleic Acid; MMP-9—Matrix Metalloproteinase 9; PD—Periodontal Disease; MS—Multiple Sclerosis; IL-2—Interleukin 2; IL-10—Interleukin 10; IL-6—Interleukin 6; MMP-7—Matrix Metalloproteinase 7; CD-90—Cluster of Differentiation 90 (Thy-1); MBP—mielin basic protein; CP-Chronic Periodontitis; TAU—Tau Protein; DJ-1—Protein Deglycase (Parkinson’s disease Protein 7).

**Table 3:** Newcastle-Ottawa scale was used to assess the eligibility of the studies selected using the criteria described by Stang et al. [37].

Main Author, Title of Research, Year of Publication	Selection				Comparability	Outcome/Exposure			Total
	C1	C2	C3	C4	C5	C6	C7	C8	
*Jakob Fehlhofer et al.,* 2024, [30]	1p	1p	1p	0p	2p	1p	1p	0p	7p
*Mohammad Hossein Mirzaii-Dizgah et al.,* 2021, [45]	1p	1p	1p	1p	1p	1p	1p	0p	7p
*Roghayeh Koshkzari et al.,* 2023, [46]	1p	1p	1p	1p	1p	1p	1p	1p	8p

C1—The exposed cohort’s representativeness (0–1 points); C2—The non-exposed cohort is chosen (0–1 points); C3—Exposure estimation (0–1 point); C4—Proof that the desired outcome was absent at the beginning of the investigation (0–1 point); C5—Cohort comparability based on design or analysis (0–2 points); C6—Evaluation of results (0–1 points); C7—Was the follow-up period sufficient for results to occur (0–1 points); C8—Proper cohort follow-up (0–1 points).

**Table 4:** The critical categories in the AMSTAR 2 [39] tool for evaluating the quality of the systematic reviews chosen for determining eligibility.

Amstar 2 Critical Criteria	*Athanasios Tsimpiris et al.* [10]	*Giovanni Schepici et al.* [9]
1. Pico elements clearly stated and research question/objective appropriately framed	Yes	Yes
2.Protocol registered before commencement of the review	Yes	Partial Yes
3. Explanation for excluded studies	Yes	Yes
4. Comprehensive literature search	Yes	Yes
5. Status of publication (i.e., grey literature) used as an inclusion criterion	Yes	No
6. List of excluded studies provided and justified	No	No
7. Risk of bias from individual studies included in review	Yes	No
8. Appropriateness of meta-analytical methods	Partial Yes	Yes
9. Consideration of risk of bias when interpreting the results	Yes	Yes
10. Assessment of presence and impact of publication bias	No	No

**Table 5:** The GRADE approach [48] is used to assess the strength of evidence from the five listed studies.

	*Fehlhofer et al.* [30]	*Mirzaii-Dizgah et al.* [45]	*Tsimpiris et al.* [10]	*Koshkzari et al.* [46]	*Schepici et al.* [9]
Year	2024	2021	2023	2023	2020
Study type	Cohort	Case-control	Meta-analysis	Case-control	Review
Initial rating	Moderate	Moderate	Low	Moderate	Low
Comparison	MS vs. controls	MS vs. controls	MS vs. controls	MS vs. controls	MS and others
Outcome	Periodontal status	MBP levels	Periodontitis prevalence	Cholinesterase activity	Biomarker identification
Study limitations (risk of bias)	Moderate	Moderate	Low	Moderate	High
Inconsistency	Not significant	Not significant	Not significant	Not significant	Significant
Indirectness of evidence	Direct	Direct	Direct	Direct	Indirect
Imprecision	Moderate	Moderate	Low	Moderate	High
Publication bias	Undetected	Undetected	Undetected	Undetected	Possible
Magnitude of effect	Moderate	Moderate	High	Moderate	Low
Dose-response association	Not applicable	Not applicable	Not applicable	Not applicable	Not applicable
All plausible biases - confounders	Yes	Yes	No	Yes	Yes
Final rating	Moderate	High	Moderate	Low	Moderate
